# Cycling in Its Relation to Health

**Published:** 1889-12-28

**Authors:** 


### HYGIENE.

### CYCLING IN ITS RELATION TO HEALTH.

The above is the title of a paper in the last *Provincial Medical Journal* by Mr. W. Armstrong Willis, of Monmouth. Mr. Willis thinks no apology necessary for introducing the subject into a medical journal, and we certainly share this opinion. For the exercise is now so general, and medical men are so often consulted as to its expediency, that it is well to have the opinion of a medical man—a cyclist himself. Dr. Willis says that about the age of thirty he was told by a medical friend "You have ceased to grow longitudinally, though not latitudinally"; and "that word latitudinally commanded his respect." After a humorous account of how he learnt the use of a machine, he asks the questions, Is tricycling safe, pleasant, useful, and easy? As for safety, accidents with moderate care are rare, and chiefly occur from turning sharp corners, going down hill too rapidly, and collisions. As for pleasantness, that depends on the roads and hills. As for utility, Mr. Willis has found his machine of great use to him in getting over his rounds, and he has often carried packages weighing over thirty pounds. At first cycling is not easy. It is a painful effort, but the first day is the worst. Facility rapidly comes with practice. The beginner should do a mile or two a day, gradually increasing the distance, and soon he will be able to get over thirty or forty miles a day "without turning a hair, without conscious effort, and without the smallest subsequent fatigue." Admitting all this, and much more advanced by Dr. Willis, we think cycling is not fitted for all sorts and conditions of human beings. Riding, on some machines at least, tends to round the shoulders and contract the chest. Healthy as such exercise on suitable machines may be for the young and strong, with unimpaired circulatory organs, it is dangerously severe exertion for persons who, advanced in life, have any heart or arterial imperfection. Especially if they mount after a meal, when serious consequences may ensue.

